# The Hidden Struggles of People Living Alone With Dementia: Loneliness and Wellbeing

**DOI:** 10.1093/geroni/igaf122.881

**Published:** 2025-12-31

**Authors:** Kate Singer, Matt Nelson, Heather Menne

**Affiliations:** Miami University, Oxford, Ohio, United States; Miami University, Oxford, Ohio, United States; Miami University, Oxford, Ohio, United States

## Abstract

The percentage of people living with dementia (PLWD) who also live alone in community settings (i.e., private residences) varies across sources. Analyses of the 2015 National Health and Aging Trends Study (NHATS) found that 16% of people with probable dementia were living alone whereas more recent analyses of the 2022 Health and Retirement Study (HRS) found that 32.5% of people 65+ years old with probable dementia were living alone. Other examinations of PLWD and people living alone with dementia (PLAwD) in the United Kingdom suggest that these populations experience higher rates of loneliness compared to the general population of older adults. The present study uses the 2022 wave of the NHATS to determine how many PLAWD are present in the sample and examines the loneliness rates among PLWD and PLAwD. The data reveals 36.0% of adults 65+ with probable dementia are living alone. Amongst PLAwD in the sample, 4.3% report feeling lonely everyday and 13.1% most days of the week. Compared to PLWD, these rates are higher, as 3.7% of PLWD report feeling lonely everyday and 3.3% most days of the week. Implications of these results are two-fold. First, there is a consistent trend that there are more people with probable dementia living alone, which begs for further programs and services for this population. Second, these findings reinforce the need to investigate ways to promote wellbeing among community-dwelling PLWD who may not have access to care partners.

